# Light scattering as an intrinsic indicator for pancreatic islet cell mass and secretion

**DOI:** 10.1038/srep10740

**Published:** 2015-06-01

**Authors:** E. Ilegems, P. P. van Krieken, P. K. Edlund, A. Dicker, T. Alanentalo, M. Eriksson, S. Mandic, U. Ahlgren, P.-O. Berggren

**Affiliations:** 1 The Rolf Luft Research Center for Diabetes and Endocrinology, Karolinska Institutet, Karolinska University Hospital L1, Stockholm SE-171 76, Sweden; 2 Umeå Centre for Molecular Medicine, Umeå University, Umeå, Sweden; 3 Diabetes Research Institute, Miller School of Medicine, University of Miami, FL 33136, Miami; 4 Lee Kong Chian School of Medicine, Nanyang Technological University, Imperial College London, Novena Campus, Singapore

## Abstract

The pancreatic islet of Langerhans is composed of endocrine cells producing and releasing hormones from secretory granules in response to various stimuli for maintenance of blood glucose homeostasis. In order to adapt to a variation in functional demands, these islets are capable of modulating their hormone secretion by increasing the number of endocrine cells as well as the functional response of individual cells. A failure in adaptive mechanisms will lead to inadequate blood glucose regulation and thereby to the development of diabetes. It is therefore necessary to develop tools for the assessment of both pancreatic islet mass and function, with the aim of understanding cellular regulatory mechanisms and factors guiding islet plasticity. Although most of the existing techniques rely on the use of artificial indicators, we present an imaging methodology based on intrinsic optical properties originating from mature insulin secretory granules within endocrine cells that reveals both pancreatic islet mass and function. We demonstrate the advantage of using this imaging strategy by monitoring *in vivo* scattering signal from pancreatic islets engrafted into the anterior chamber of the mouse eye, and how this versatile and noninvasive methodology permits the characterization of islet morphology and plasticity as well as hormone secretory status.

The islets of Langerhans in the pancreas are secreting hormones in response to diverse stimuli in order to maintain proper blood glucose homeostasis. In particular the beta cells produce insulin, which is stored as dense crystals in cytoplasmic secretory granules^1^. Insulin is released into the blood stream as a consequence of increased glucose levels and thereby ensuring maintenance of blood glucose homeostasis. In response to varying functional demands, islets are required to modulate their secretory activity and/or alter the number of endocrine cells they are composed of. An inadequate islet mass and function will lead to the inability to control blood glucose concentration, and eventually to diabetes. It has therefore been of major interest to develop methods for the assessment of pancreatic islet cell mass and function for the understanding of regulatory mechanisms at the cellular and organism level.

So far a number of techniques have been developed and applied for the determination of either pancreatic islet mass or islet function. For instance islet mass has been studied by bioluminescence imaging^2^, fluorescence imaging^3^,^4^, magnetic resonance imaging^5^, positron-emission tomography^6^, optical projection tomography^7^, or optical coherence microscopy^8^. Various probes have been developed for islet function, assessing e.g. glucose uptake and metabolism^9^,^10^,^11^, cytoplasmic free Ca^2+^concentration^12^, or insulin secretion^13^. Many of these methodologies require the use of heterologously-expressed biomarkers or the administration of extrinsic labels, potentially affecting normal cellular functions. Here we report on an intrinsic indicator for both islet mass and function, based on scattering properties of pancreatic islets. We show that three-dimensional information of islet morphology can be acquired without the use of artificial indicators, both *in vitro* and *in vivo*. Additionally, we demonstrate that scattering properties of pancreatic islets depend on the abundance of hormone-containing mature secretory granules, enabling longitudinal observations of the secretory status of individual islets.

## Results

### Pancreatic islets can be visualized using their intrinsic light scattering properties

**Figure 1:**
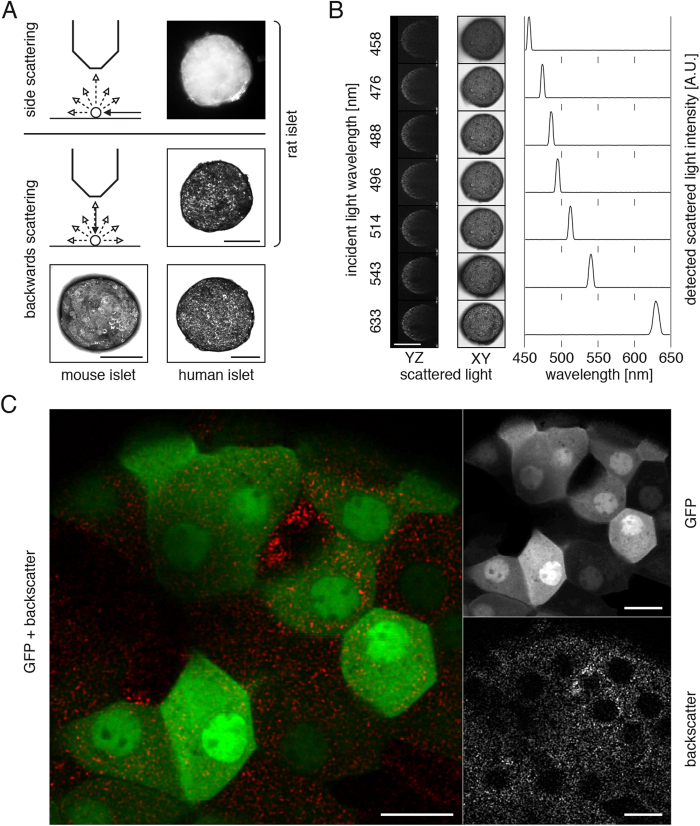
Pancreatic islets can be visualized using their intrinsic backscattering properties. (**A**) Intrinsic scattering properties of pancreatic islets from various species allow their visualization *in vitro* by imaging side scattering of light by optical microscopy and backwards scattering of a laser beam scanning by confocal microscopy. This latter imaging technique not only offers high-resolution imaging but also provides three-dimensional information by the imaging of optical slices. (**B**) Different laser illumination wavelengths can be used to acquire backscatter images, the maximum signal intensity being collected at the incident light wavelength. (**C**) Confocal imaging of MIP-GFP islets at higher magnification reveals a punctuated pattern of backscatter signal, excluded from cell nuclei. The backscatter signal is present non-exclusively in each GFP-positive cell. Confocal images are presented as maximum intensity projections (**A**, **B**) or single optical planes (**C**). Size bars = 100 μm (**A**, **B**), 10 μm (**C**).

Pancreatic islets originating from different species have been qualitatively assessed for their light scattering properties (Fig. 1A). The islet morphology can be visualized at various scattering angles, for instance using an incident white light at an angular position of 90° relative to the observation microscope, or using an incident laser beam at an angular position of 180° for imaging backscattered signal by confocal microscopy. Backscatter signal provides identical three-dimensional morphological information at various laser illumination wavelengths, with a unique detection signal peaking at the incident light wavelength (Fig. 1B).

To assess whether backscatter signal originates from beta cells we imaged pancreatic islets isolated from MIP-GFP transgenic mice at high resolution by confocal microscopy. We observed that the backscatter signal forms a punctuate pattern, excluded from cell nuclei. Since beta cells within these particular islets are expressing the green fluorescent protein EGFP^4^, we could determine that all beta cells possess intrinsic backscatter properties – albeit non-exclusively (Fig. 1C).

### Backscatter signal permits the quantification of individual islet volumes without cellular staining

**Figure 2:**
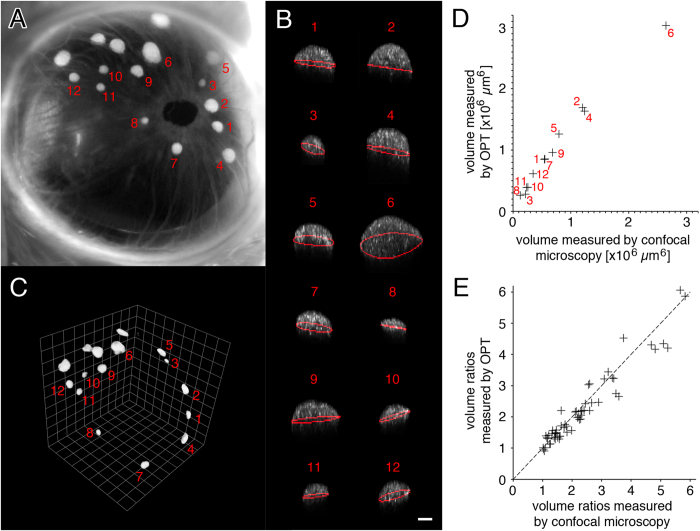
Backscatter signal can be used as an intrinsic indicator for islet volume. (**A**) Photography of a transplanted eye shows a number of individual islets of various dimensions. (**B**) The islets were analyzed individually by computational analysis after *in vivo* imaging (islets represented as maximum intensity projection on XZ; white is backscatter signal; red line is equatorial area). **(C)** Mice were then sacrificed and their eyes collected, processed using an anti-insulin antibody for staining, and scanned by optical projection tomography (OPT) to determine individual islet volumes. (**D**) Comparison of individual islet volumes as quantified from images obtained *in vivo* by confocal microscopy and from images obtained *ex vivo* by OPT. (**E**) The ratios between individual islet volumes from islet grafts in three different mice are plotted and shown to be independent of the imaging technique in use. Size bar = 50 μm.

In order to image individual islets non-invasively *in vivo*, we have isolated pancreatic islets from donor mice and proceeded with syngeneic transplantations into the anterior chamber of the eye of recipient mice using a technique previously established^14^,^15^. The islet grafts can then be imaged through the transparent cornea in the living animal under anesthesia (Fig. 2A). Three-dimensional information of individual islets was acquired by backscatter imaging and their respective volumes were determined by computational image analysis (Fig. 2B). In order to validate our image analysis protocol based on the backscatter signal collected from islet grafts *in vivo*, we next pursued an *ex vivo* analysis of islet mass by optical projection tomography (OPT^16^). The transplanted eye was collected, processed and stained using an anti-insulin antibody before imaging. Visualization of the insulin-positive signal from the eye allowed for the identification of the same islet grafts previously imaged *in vivo* (Fig. 2C). The individual islet volumes obtained by *in vivo* imaging compared to OPT scanning indicate a linear correlation (Fig. 2D). Because of technical differences between both imaging methodologies, such as changes in tissue volume after *ex vivo* processing and image analysis protocols, the volumes obtained are not identical. However the ratios between individual islet volumes proved to be identical regardless of the imaging technique in use, validating backscatter imaging as a tool to non-invasively monitor differences in islet cell mass (Fig. 2E).

### Backscatter imaging allows monitoring and characterizing islet growth *in vivo*

**Figure 3:**
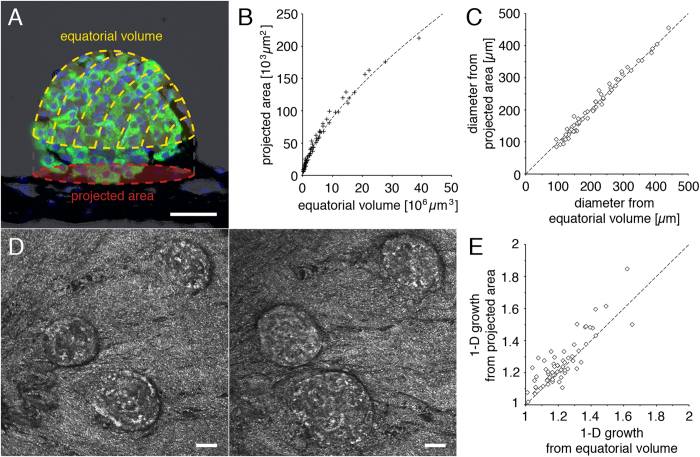
Quantification and characterization of islet growth *in vivo* by backscatter imaging. (**A**) A schematic representation of dimensional information obtained by *in vivo* islet backscatter imaging is overlaid on an islet graft section stained by immunohistochemistry (green = insulin; blue = DAPI; black = pigmented iris). *In vivo* imaging permits longitudinal analysis of the islet equatorial volume and projected area. (**B**) Equatorial volume and projected area from individual islets imaged *in vivo* and (**C**) calculated diameter based on a spherical model. (**D**) *In vivo* imaging of the same islets engrafted into the anterior chamber of the eye at two different time points allows the longitudinal assessment of islet growth in the ob/ob mouse. (**E**) Growth of 27 islets was assessed over a period from 1 to 3 months in 7 ob/ob mice. The average X-, Y-, or Z-growth based either on the equatorial volume or on the projected area shows no preference for directional growth in the ob/ob mouse model. Size bars = 50 μm.

We visualized pancreatic islets engrafted into the anterior chamber of the mouse eye *in vivo* by backscatter imaging, providing both three-dimensional volumetric information from islet image modeling and two-dimensional information from the projected area (Fig. 3A,B). Interpolation of volumetric and planar data to diameters of a sphere provides similar values, demonstrating the highly spherical nature of islet grafts (Fig. 3C).

Next we investigated the possibility to not only assess three-dimensional volumetric differences between distinct islet grafts, but also to evaluate differences in the morphology of the same islet graft at different time points. Leptin-deficient obese mice were transplanted into the anterior chamber of the eye, and their islet grafts imaged longitudinally over a period of one to three months. The particularly high beta cell proliferation in these mice^17^ results in a distinct growth of engrafted islets (Fig. 3D).

To assess whether this growth occurs homogenously in all spatial directions we quantified both the equatorial volumes and projected areas of individual islets at different imaging dates, and interpolated these values to the radii of model spheres. The ratio between one-dimensional growth obtained from the equatorial volume and that obtained from the projected area is equal to 0.97 ± 0.06, indicating that growth of islet grafts does not occur preferentially in one direction but is homogenous in all directions in the ob/ob mouse model (Fig. 3E).

### Backscatter signal in pancreatic islets originates primarily from zinc-containing granules and indicates hormone secretory status

**Figure 4:**
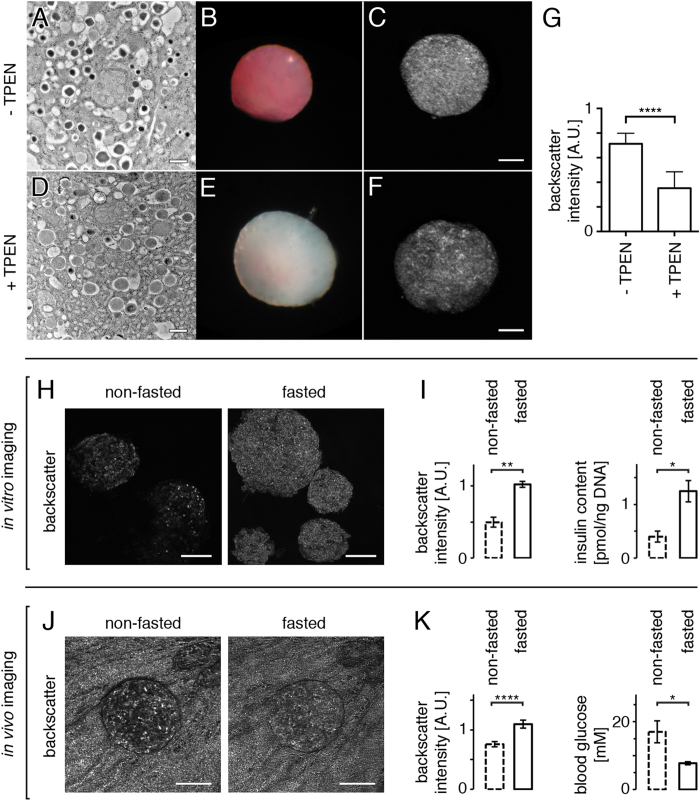
Backscatter intensity in beta cells originates from dense core secretory granules and provides information on islet secretory status. (**A**) Electron microscopy imaging shows that untreated isolated pancreatic islets display typical dense core secretory granules containing zinc-insulin crystals. (**D**) The chelation of Zn^2+^ by treatment with TPEN impedes with normal crystallization, resulting in pale granules. (**B**, **E**) Dithizone staining in treated versus non-treated islets demonstrates a clear loss of Zn crystals after TPEN treatment. (**C**, **F**, **G**) The reduction in the number of dense core granules results in a decreased backscatter signal intensity. (**H**) Islets were isolated from mice having either free access to food or been food-deprived for 12 h, and their backscatter signal was acquired by confocal microscopy. (**I**) Both backscatter signal intensity and insulin content were increased when mice were fasted overnight. (**J**) *In vivo* imaging of an islet engrafted into the anterior chamber of the ob/ob mouse eye on two consecutive days shows differences in backscatter signal intensities depending on whether the mouse had free access to food or had been food-deprived for 12 h prior to imaging. Note that the backscatter signal originating from the strongly reflective pigmented iris is similar in both images, illustrating in this case that under identical imaging conditions the variations observed in signal intensities are islet-specific. (**K**) Quantification of backscatter signal intensities *in vivo* shows an increase under fasting conditions, correlating with lower blood glucose levels resulting in decreased insulin secretion from islets (n = 19 islets in 3 mice). Values are average ± SEM. ^*^P < 0.05; ^**^P < 0.01; ^****^P < 0.0001. Size bars = 100 μm.

Beta cells contain thousands of secretory granules enclosing dense-core crystals, which represent about 10–20% of the cellular volume^18^,^19^,^20^,^21^. These granules being in a size range of 200–400 nm, which is similar to the wavelength of light used in our setup for backscatter imaging, we hypothesized that the highly reflective nature of pancreatic islets is due to Mie scattering from these dense core granules. We thus investigated the effect of a reduction in insulin crystallization within the granules by depriving pancreatic islet cells from Zn^2+^, an essential ion for the formation of insulin crystals^22^. We treated isolated islets with N,N,N′,N′-tetrakis(2-pyridylmethyl)ethylenediamine (TPEN), a Zn^2+^chelator, removing intracellular Zn^2+^and impeding with the novel formation of insulin crystals within secretory granules. Eight hours post-treatment, insulin molecules within granules fail to crystallize, as seen by electron microscopy (Fig. 4A,D). Dithizone staining was also impaired in the treated samples (Fig. 4B,E). The fact that dithizone staining is specific to zinc-insulin crystals^23^ further confirms a reduction in the density of secretory granules density, correlating with the decreased backscattering intensity of TPEN-treated islets (Fig. 4C,F,G). The analysis of average backscatter signal intensity originating from individual beta cells within the islet (as identified using MIP-GFP islets as for Fig. 1C) also showed a strong decrease following treatment with TPEN, demonstrating the contribution of insulin-containing secretory granules to the overall islet reflective property (not shown).

Since a fraction of the mature insulin containing granules are involved in the secretory process subsequent to the increased blood glucose concentration following food consumption^24^, we compared the average backscatter signal intensity of islets partially degranulated to that of islets under low secretory demands. We isolated pancreatic islets from mice that were either fed or fasted overnight and measured *in vitro* that both backscatter signal intensity and insulin content were increased when mice were food-deprived before isolation, due to a lower insulin secretion (Fig. 4H,I). Similarly, when imaging *in vivo* individual pancreatic islets engrafted into the anterior chamber of the mouse eye on two consecutive days, we measured differences in backscatter signal intensities depending on whether mice had free access to food or were food-deprived prior to imaging (Fig. 4J,K). The backscatter signal thus not only serves as an intrinsic indicator of pancreatic islet cell mass and morphology but also can report on the islet secretory status, both *in vitro* and *in vivo*.

## Discussion

The scattering of light is involved in a decreased penetration of light through samples^25^, normally a detrimental factor both for optical imaging and electromagnetic radiation therapy. It has however also been used in the past as a valuable tool for imaging or detecting samples^16^,^26^,^27^ and here for imaging pancreatic islets by laser scanning confocal microscopy. The presented methodology relies on the particular reflective properties of islet cells originating from various species, and offers imaging versatility in terms of optical detection wavelength. We have developed an image analysis protocol allowing not only to quantify individual islet volumes but also to monitor dynamic changes in islet cell mass and morphology. This protocol was cross-validated with an established imaging technology, indicating that quantification of islet volume obtained by confocal backscatter imaging correlates with that of beta cell mass by optical projection tomography. Combining backscatter imaging with *in vivo* imaging of tissue transplanted into the anterior chamber of the eye, we could use this methodology to noninvasively characterize changes in beta cell mass and islet morphology in an animal model of type 2 diabetes.

Mie scattering of a ray of light can occur on particles of similar dimension to that of the incident ray of light’s wavelength^28^. While all cells display some level of Mie scattering mainly due to mitochondria^29^, we show here that the physical origin of backscattering in beta cells originates primarily from mature insulin secretory granules. The biogenesis of these granules starts with the synthesis of pro-insulin, the formation of pale immature progranules, assembly of pro-insulin-zinc hexamers that will finally be processed into zinc-insulin hexamer crystals tightly packed into mature granules^1^,^30^. As much as about 10-20% of the volume of beta cells is constituted by granules (compared to 4% by mitochondria), explaining the strong reflective properties of these particular endocrine cells. By chemically reducing intracellular Zn^2+^concentrations we could interfere with the formation of mature granules, and instead observed pale immature granules of low scattering properties. Interestingly it has been shown previously that a decrease in intragranular Zn^2+^concentration by removal of the zinc transporter ZnT8 similarly leads to the presence of pale progranules instead of dense core mature granules in beta cells, and that these islets are characterized by a more transparent appearance^23^,^31^. This supports our finding that a reduced abundance of zinc-insulin crystals correlates with a reduction in backscatter signal intensity, and thus that the particular scattering properties of beta cells is directly dependent on the presence of mature insulin granules.

It implies that optical properties of pancreatic islets can be used to assess their level of degranulation in response to stimuli, which we could demonstrate by the assessment of different levels of insulin secretion *in vivo* in the same animal. We witnessed a degranulation in response to food intake regardless of whether the islets originated from the pancreas or if they were engrafted into the anterior chamber of the eye, illustrating that we can assess day-to-day differences in the scattering properties of islets due to their varying hormone secretory status and depending for instance on feeding behavior.

Interestingly, while we can observe insulin secretory status at the islet level, backscatter images obtained at single cell level display an evident heterogeneity in terms of insulin expression and secretion among endocrine beta cells within the islet. It has indeed been shown that beta cells can be categorized into subpopulations having different thresholds in response to stimuli, leading to diverse levels of degranulation^32^,^33^,^34^. Backscatter imaging is thus a versatile and noninvasive technique that can serve as a tool to investigate the endocrine part of the pancreas from the islet population to the single cell level, both for the understanding of molecular mechanisms regulating their functional plasticity and for the development of novel therapeutic strategies for the modulation of beta cell mass and secretion.

## Materials and Methods

### Chemicals

All chemicals are from Sigma-Aldrich, unless otherwise specified.

### Animal models, human tissue, and blood glucose measurements

C57BL/6J and SJL mice were from Charles River (Germany). MIP-GFP mice^4^, backcrossed with C57BL/6J mice, and ob/ob mice are inbred at the animal core facilities at the Karolinska Institutet. Wistar rats (10 weeks-old) were obtained from Charles River (Germany). Human pancreatic islets were obtained within the Nordic Network for Islet Transplantation from deceased donors. Blood glucose concentrations in mice were obtained using Accu-Chek Aviva monitoring system (Roche). All experiments were performed following Karolinska Institutet’s guidelines for care and use of animals in research and were approved by the animal ethics committee at Karolinska Institutet.

### Pancreatic islet isolation and transplantation

Pancreatic islets were prepared and purified from donor animals following standard procedures^35^, maintained in RPMI 1640 medium supplemented with 10% fetal bovine serum (Life Technologies), and kept at 37 °C in a humidified atmosphere with 5% CO_2_ for a maximum of 2 days before use. Animals were transplanted into the anterior chamber of the eye at 4–8 weeks of age with islets isolated from age-matched donors, as described previously^14^. During transplantation isoflurane was used for anesthesia, and mice received subcutaneous injections of Temgesic (Schering-Plough) for post-operative analgesia.

### Laser Scanning Confocal Microscopy

Laser scanning confocal micrographs were recorded using a upright TCS-SP2-AOBS (Leica) equipped with 10x, 20x, and 40x water-immersion objectives. Fluorescence emission from GFP was obtained by excitation at 488 nm and detection between 500 and 550 nm. For consistency reasons, backscatter signal imaging was obtained using a 633 nm laser beam for all figures unless otherwise specified, with detection at the same wavelength as the incident laser light. *In vivo* imaging of islet grafts in mice anesthetized with isoflurane was performed as previously described^14^, 2–5 months after transplantation. Scanning speed and laser intensities were adjusted to avoid any cellular damage to islets *in vitro*, and to the mouse eye or islet graft *in vivo*. All confocal images are representatives and presented as maximum intensity projections in the XY plane unless otherwise stated.

### Transmission electron microscopy (TEM)

Pancreas was dissected and small pieces were fixed in 2,5% glutaraldehyde +1% paraformaldehyde in 0.1 M phosphate buffer, pH 7.4 at room temperature for 30 min and stored in fixative at 4 °C. Specimens were rinsed in 0.1 M phosphate buffer, pH 7.4 and postfixed in 2% osmium tetroxide 0.1 M phosphate buffer, pH 7.4 at 4 °C for two hours, dehydrated in ethanol followed by acetone and embedded in LX-112 (Ladd, Burlington, Vermont, USA). Semi-thin sections were cut and stained with toluidine blue O and used for light microscopic analysis. Ultrathin sections (approximately 50–60 nm) were cut by a Leica EM UC 6 (Leica, Wien, Austria) and contrasted with uranyl acetate followed by lead citrate and examined in a Tecnai 12 Spirit Bio TWIN transmission electron microscope (FEI company, Eindhoven, The Netherlands) at 100 kV. Digital images were taken by using a Veleta camera (Olympus Soft Imaging Solutions, GmbH, Münster, Germany).

### Immunohistochemistry

Paraffin sections of mouse tissue were prepared and processed as previously described^14^. Imaging of slides was performed using a BD Pathway 855 system (BD Biosciences).

### Insulin measurements

Insulin concentrations were obtained using mouse insulin ELISA plates (Mercodia), and normalized to islet DNA content measured using Quant-iT PicoGreen dsDNA assays (Life Technologies).

### Optical Projection Tomography (OPT)

SJL mice were used for OPT scanning and cross-validation of imaging techniques. One month after syngeneic transplantation of pancreatic islets into the anterior chamber of the eye, islet grafts were imaged *in vivo* by confocal microscopy under anesthesia before euthanasia. The eyes were then collected and fixed for 2 hours with 4% paraformaldehyde on ice. Immunolabeling, using anti-insulin primary antibody (DAKO) and Alexa Fluor 594 labeled secondary antibody (Life Technologies), and tissue processing for OPT scanning, using the Bioptonics 3001 OPT scanner (Bioptonics), was performed as described previously^16^. OPT scanning and reconstruction was performed using an established procedure^36^, islet quantification was performed using Imaris and image rendering using Volocity.

### Image processing and analysis

AutoQuant X2 (Media Cybernetics, Bethesda, MD) was used for blind deconvolution of all confocal images. The analysis of islet volume based on backscatter signal obtained by confocal microscopy was performed using Matlab (Mathworks). The “equatorial volume” of islets is defined as the volume from the top of the islet down to the calculated equator of a spheroid model based on a three-dimensional reconstruction of backscattered light. The backscatter signal intensity of each islet was quantified by averaging voxel intensities in a cylinder having a length of 50 μm from the top towards the center of the islet, and a diameter being half of that of the islet. For OPT-generated images, iso-surface reconstructions and quantification of islet volumes were obtained using Volocity (Perkin Elmer). Volocity was used for image display and Adobe Photoshop for image assembly.

## Additional Information

**How to cite this article**: Ilegems, E. *et al.* Light scattering as an intrinsic indicator for pancreatic islet cell mass and secretion. *Sci. Rep.* **5**, 10740; doi: 10.1038/srep10740 (2015).
